# Improving the design of nutrition labels to promote healthier food choices and reasonable portion sizes

**DOI:** 10.1038/ijo.2014.86

**Published:** 2014-07-25

**Authors:** C A Roberto, N Khandpur

**Affiliations:** 1 Department of Social and Behavioral Sciences, Harvard School of Public Health, Cambridge, MA, USA; 2 Department of Nutrition, Harvard School of Public Health, Boston, MA, USA

## Abstract

Accurate and easy-to-understand nutrition labeling is a worthy public health goal that should be considered an important strategy among many to address obesity and poor diet. Updating the Nutrition Facts Panel on packaged foods, developing a uniform front-of-package labeling system and providing consumers with nutrition information on restaurant menus offer important opportunities to educate people about food's nutritional content, increase awareness of reasonable portion sizes and motivate consumers to make healthier choices. The aims of this paper were to identify and discuss: (1) current concerns with nutrition label communication strategies; (2) opportunities to improve the communication of nutrition information via food labels, with a specific focus on serving size information; and (3) important future areas of research on nutrition labeling as a tool to improve diet. We suggest that research on nutrition labeling should focus on ways to improve food labels' ability to capture consumer attention, reduce label complexity and convey numeric nutrition information in simpler and more meaningful ways, such as through interpretive food labels, the addition of simple text, reduced use of percentages and easy-to-understand presentation of serving size information.

## Introduction

In the past four decades, obesity in both adults and children has increased dramatically.^1,2^ The rapid rise is thought to be due largely to changes in the food and physical activity environments, given the relative stability of the population's gene pool over this time. Energy-dense, nutrient-poor foods are conveniently available and heavily marketed.^3, 4, 5, 6, 7^ In addition, the past two decades have seen a proliferation of restaurants, increased snacking, decreased family meals and greater consumption of meals prepared outside the home.^8, 9, 10, 11^ The growth in portion sizes of packaged and restaurant food have been implicated in increasing obesity prevalence.^12,13^ Portions of French fries, hamburgers and sugar-sweetened beverages have more than doubled in size,^12,14^ and a robust body of research has found that people consume more when served larger portions.^15, 16, 17^

The USDA's 2010 Dietary Guidelines advise Americans to control total caloric intake and reduce sodium, saturated fat, *trans* fat, cholesterol and added sugar consumption.^18^ The provision of clear and accurate nutrition information is one important way to help consumers adhere to these guidelines and make informed choices. Nutrition labels on food packaging and restaurant menus offer one of the best ways to disseminate and make salient such information at the point-of-purchase, when it is arguably most likely to influence purchasing behavior. In addition, required disclosure of nutrition information can incentivize food manufacturers to improve the nutrient profile of their products.^19^

Recent global food policy efforts have focused on providing consumers with greater access to easy-to-understand nutrition information. In the United States, the Food and Drug Administration (FDA) has expressed interest in updating the Nutrition Facts Panel (NFP) on packaged foods to improve its clarity^20^ and undertook an initiative^21^ to recommend a uniform, front-of-package (FOP) labeling system that could be adopted by the food and beverage industries.^22,23^ In addition, a menu labeling mandate, included as part of the 2010 Patient Protection and Affordable Care Act,^24^ will require chain restaurants with ⩾20 locations to provide calorie information on restaurant menus at the point-of-purchase.

Although a growing number of studies have examined effective ways to communicate nutrition information through the NFP and newer labeling initiatives, there is still much to learn. In addition, fewer studies have focused specifically on educating consumers about appropriate serving and/or portion sizes via nutrition labels. Therefore the aims of this paper were to identify and discuss: (1) current concerns with nutrition label communication strategies; (2) opportunities to improve the communication of nutrition information via food labels, with a specific focus on serving size information; and (3) important future areas of research on nutrition labeling as a tool to improve diet. In this paper, serving size refers to the amount of a food recommended for consumption in one sitting, while portion size refers to the actual amount of food a person portions out for consumption in one sitting.^25^ Portion size and serving size are related concepts, but they exert different influences on the amount of food consumed. In this paper, we discuss the ways in which serving size information can influence consumer perceptions of appropriate portion sizes, which in turn influence the amount consumed.^13,14^

## The NFP on packaged foods

The passage of the Nutrition Labeling and Education Act of 1990 required the provision of standardized nutrition information through the NFP on most packaged foods in the United States.^26^ Although some nutrition information on the NFP can vary based on the food product, the standard label includes information about serving size, kilocalories (kcal; calories) and calories from fat, total, saturated and *trans* fat, cholesterol, sodium, total carbohydrates, dietary fiber, sugars and protein. The NFP also displays information for certain vitamins and minerals. Nutrient amounts are presented in grams and milligrams accompanied by percentages derived from recommended daily allowances or daily values (based on a single serving for a 2000 kcal diet).^26,27^

### Consumer use of the NFP

Across studies, approximately half of American adults report using the NFP when making food-purchasing decisions, suggesting it is an important source of information for consumers.^28, 29, 30, 31^ More specifically, 54% of adult respondents in the 2008 Health and Diet Survey reported using the NFP ‘often' when buying a product for the first time, and >60% reported they ‘often' or ‘sometimes' accessed information about calories and serving size.^28^ In a similar sample of adults, 53% reported using the NFP ‘always or almost always' when making food-purchasing decisions.^29^ However, objectively measured viewing of the NFP with eye-tracking technology suggests these self-reported estimates of label usage may be inflated.^32^ Graham and Jeffery^32^ found that only 9% of 203 adult participants viewed the NFP calorie content during a food purchasing task, despite 33% self-reporting that they ‘almost always' used it when food shopping.^32^ Similarly, although 31% reported ‘almost always' looking at the total fat content on the NFP, the eye-tracking data revealed that <1% actually did.

Although intended for use by the entire population, nutrition labels are more likely to be used by those who are well-educated, Caucasian, female and/or young adults^33^ as well as by those with healthier eating habits, higher incomes and greater nutrition knowledge.^33,34^ A greater proportion of non-NFP users tend to be overweight, Black or Hispanic, unmarried and male.^29^ Unfortunately, it is not uncommon to find low NFP use among population groups who stand to benefit most from it.^35^ Design limitations of the current NFP might partially explain why it is an under-utilized source of nutrition information. There is, however, evidence that people with chronic disease (that is, hypertension, diabetes, heart disease) report greater nutrition awareness and food label use compared with those without chronic disease.^36^

### Addressing concerns about the NFP

#### The problem of serving size label inconsistencies

All of the nutrient information presented on the NFP hinges upon the listed serving size. Serving size labels are created by food manufacturers based on Reference Amounts Customarily Consumed (RACC) Per Eating Occasion described in common household measurements appropriate to the type of food.^26,37,38^ The RACC were originally determined by the FDA based on Nationwide Food Consumption Surveys conducted in the late 1970s and 1980s. In instances when survey data were inadequate, other sources were considered, including dietary guidance recommendations and serving sizes used by manufacturers, grocers and other countries. One concern with continuing to use the RACC is that stated serving sizes of commonly consumed items, such as cereal and punch, have been found to be substantially less than what is realistically portioned out by consumers.^39,40^

Another concern is that serving sizes for packaged food can vary over a wide range. Current guidelines state that one unit of a food commodity can be considered a single serving if it weighs between 67% and 200% of the RACC.^37^ Usually the nutrition information for a food containing ⩾200% of the RACC is based on a single RACC serving, and the package indicates the number of servings it contains. However, a packaged food container weighing >200% of the RACC can also be considered a single serving if the food manufacturer believes that the entire container can reasonably be eaten during a single eating occasion.^38^ Although in 2004 the FDA encouraged food manufacturers to label foods usually consumed in one sitting as a single serving, there has not been a formal mandate to do so.^26^ Given the increase in portion sizes over time, it is unclear whether the RACC should also be overhauled to reflect what consumers are actually eating or if an increase in the RACC would inadvertently promote further overconsumption; these questions warrant further study.

The current FDA guidelines allow food manufacturers flexibility to define the amount of a single serving of their product.^41^ This means that two very similar products could appear to have different nutritional profiles depending on the serving size.^41^ For example, Mohr *et al.*^41^ identified that the RACC for a regular candy bar (typically consumed in one sitting), is 40 g. At the time, they found that this was the serving size listed for the Endangered Species Brand Milk Chocolate and Peanut Butter bar. However, one serving of a 3 Musketeers Bar and a Milky Way Bar was listed as 23 g (57.5% of the RACC), and thus a serving of those candy bars appears to be half the calories of a serving of the Endangered Species bar.^41^ These kinds of discrepancies in serving size within the same food category have been documented for products, such as granola bars, yogurt, soup and candy bars.^41^

Mohr *et al.*^41^ call this kind of serving size manipulation ‘health framing', because consumers who view items with smaller serving sizes are prone to incorrectly perceive the product as healthier than a comparable product with a larger serving size. In one study, Mohr *et al.*^41^ randomized 151 participants from an Internet panel to view a pizza and soup product where the unit weight of the product and product serving sizes were manipulated. The study revealed that health framing (presenting smaller serving sizes) reduced the anticipated guilt of consuming the product and increased the intent to purchase the product. This effect was moderated by level of dietary concerns; health framing led those with high dietary concerns to experience significant reductions in anticipated guilt. This suggests that those most concerned with nutrition, and therefore more likely to read the NFP, might also be most vulnerable to the negative effects of health framing. The results from this study indicate that health framing might influence consumers at the point-of-purchase to buy a specific product or choose that product over similar ones. However, it is unknown whether such health framing impacts consumers at the point of consumption. It is possible that the smaller serving size advocated on the packaging influences consumers to eat less. Alternatively, the health frame might create an initial health halo that persists, which could translate into greater consumption;^42^ these are important questions for future research to address.

#### Concerns about consumer numeracy and literacy

National and international surveys have found that >90 million Americans have limited literacy skills,^43^ which raises concerns about the amount of numeric and technical information on the NFP. Several studies have documented consumer difficulty understanding quantitative information presented on food labels, especially with respect to serving size information^44, 45, 46^ and the percentages of recommended daily amounts.^47, 48, 49, 50^

#### Serving size calculations

In one study, portion size estimation skills of primary care patients were assessed by asking them to serve an amount of three foods and one beverage that represented what they thought a single standard serving was for each of the products. Then patients were told what the actual serving size was and were asked to serve that specific amount. The sample consisted primarily of women, half of whom reported having previous nutrition and portion size education. The results revealed that higher literacy (but not numeracy) was associated with greater accuracy when portioning out a single serving of the foods/beverage.^44^ In another study of 90 health center patients, 86% of the respondents assumed that a unit of packaged food was a single serving even if it contained multiple servings and incorrectly equated calories from a single serving with the caloric content of the entire package.^45^ After some assistance and prompting from research staff to re-evaluate incorrect answers, people improved only slightly, with 63% of the participants still confusing calories per serving with total calories in the package. Participants in this study who had low levels of education were more prone to incorrectly apply information from the NFP to estimate calories contained per package.^45^

A study conducted by Rothman *et al.*^46^ also examined patients' ability to read and understand nutrition information on food labels. Only 32% of patients could accurately calculate the number of carbohydrates that would be consumed when drinking a 20-oz. bottled beverage containing 2.5 servings. Only 22% of patients could determine total carbohydrates when presented with nutrition information for two slices of low-carb bread.^46^ Across study tasks, people consistently made errors when trying to mathematically manipulate serving size information to draw a conclusion about a food's nutritional profile. In this study, low numeracy and literacy skills were also significantly associated with poor understanding of nutrition labels. Finally, another study found that as little as 10.5% of college students could correctly describe serving size from the NFP after viewing different food labels.^51^ The results from these studies highlight the difficulty people have manipulating and using the numeric information presented on labels with respect to serving size, particularly for foods containing multiple servings.

#### Addressing serving size inconsistencies through labeling

One proposed way to address the confusion around serving size is to change the NFP design to include two columns: one that contains nutrition information for a single serving and one that contains nutrition information for the entire container, particularly if it is a packaged food or beverage typically consumed in one sitting.

A study by Antonuk and Block^52^ randomized undergraduate students to either a single- or dual-labeled NFP appearing on a package of 50 M&Ms they could eat while watching a short video. The study found that non-dieters exposed to the dual-column NFP reduced their consumption of M&Ms; dieters M&M consumption did not differ between groups. Although dieters ate significantly fewer M&Ms compared with non-dieters when exposed to the single-column label, the dieters and non-dieters in the dual-column group did not significantly differ in the amount of M&Ms consumed. These results suggest that the dual-label column approach has the potential to encourage healthier eating behavior.

In addition, Lando and Lo^53^ conducted an online study examining the dual-column NFP format. Approximately, 9500 participants recruited from an Internet panel were randomized to one of the 40 study arms. The study used a 10 (label format) × 2 (product category: frozen meal or a bag of chips) × 2 (healthy versus less healthy food) design. The tested labeling formats presented nutrition information as either: (i) two servings per container with nutrient information listed per serving in a single column (five different versions), (ii) two servings per container with a dual column: one column listing nutrient information per serving and the other listing information per package (three different versions), or (iii) one serving per container, with nutrient information listed per serving in a single column (two different versions). The different label versions also involved the removal of calories from fat and/or enlarged font for calories. The current NFP, with two servings per container, served as the control.

Results revealed that participants rated products as less healthful when they were labeled with one serving per container. Relative to the current NFP, participants could more accurately determine the nutrient content of a product when it was labeled with a single column containing one serving or when information was presented in dual columns (per serving information in the first column and information per package in the second column). When products had the same NFP format, there were no significant differences in participants' ability to select the more healthful of the two products. However, when comparing products with different NFP formats, the greatest proportion of participants could accurately identify the more healthful product (75%) and calories per container (68%) when a dual-column label was compared with a two servings, single-column label (the current NFP format). Enlarging the font size for calories and removing ‘calories from fat' did not independently affect label usability.

The findings from these two studies suggest that the addition of a second column presenting nutrient and calorie information for an entire package, rather than per serving, might be more helpful for the consumer. However, such a format would mean adding more information to an already complex and busy label. Therefore, the option of a single column for products typically consumed in one sitting, with the serving size based on the entire package, might be preferred. However, before adopting this new labeling scheme for the NFP, additional research should compare the dual- and single- column labels to even simpler presentation formats that provide less information and use creative methods to interpret the information for the consumer, including Traffic Light labels or other graphical displays. Such labeling schemes must also be tested during real-world shopping trips.

#### Reducing the amount of complicated information on the NFP

Taylor and Wilkening^26^ explain that great care was taken when designing the original NFP to consider research ‘about comprehension, legibility, and literacy, taking into account the needs of the elderly and others with sight limitations.'^54^ For example, specific design elements were added to improve usability, such as the inclusion of lines between nutrients, the removal of punctuation marks, the use of larger type and upper and lower case letters, instead of only uppercase, and the bolding of important nutrient information. The NFP is also displayed in a box with a white background to make it stand out from the food packaging.

Although designed to be easy-to-use, infrequent use of the NFP, particularly by certain demographic groups, might be partially explained by the large amount of complicated information presented on the label. In Graham and Jeffery^32^ eye-tracking study, most consumers typically only viewed the top five lines of the label, suggesting that much of the additional information may rarely get read, except perhaps by highly nutrition-conscious consumers. The bottom half of the label also presents additional information about grams/milligrams of nutrients based on a 2000 versus 2500 kilocalorie diet. As the FDA discusses altering the NFP to improve usability, it would be worth considering whether all of this information should remain or if a better approach is to include less but more meaningful and salient information. The small font of the NFP has also been cited as a deterrent to its use.^34^

**Figure 1:**
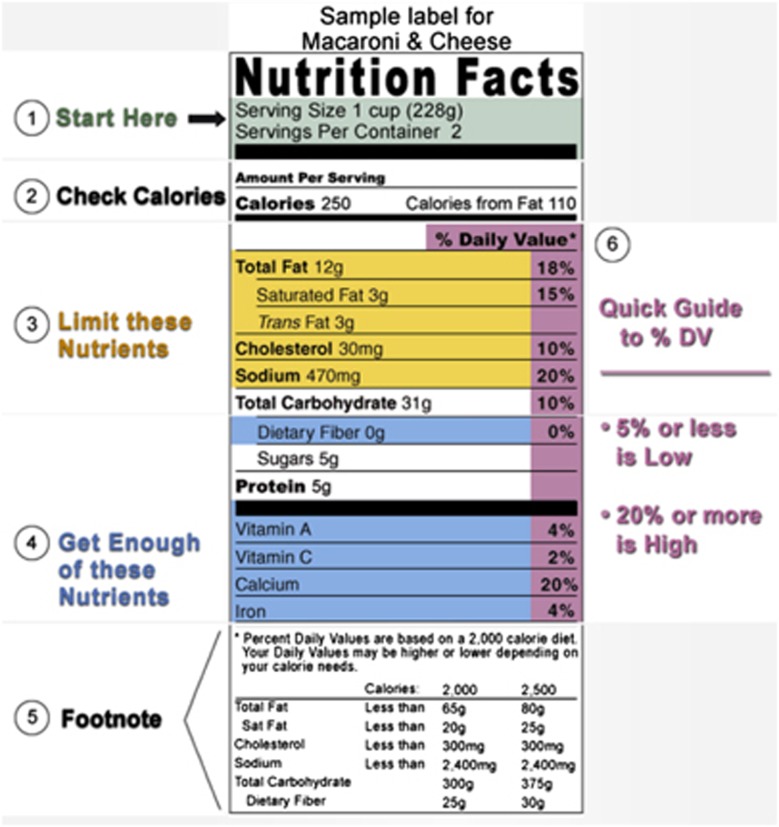
The Nutrition Facts Label overview presented on the US Food and Drug Administration website. Republished here with permission from the US Food and Drug Administration. http://www.fda.gov/. Available from: http://www.fda.gov/food/ingredientspackaginglabeling/labelingnutrition/ucm274590.htm. Accessed June 2013.

Another concern with the NFP is the use of percentages, which were originally included to put the nutrition information in the context of an overall daily diet and enable easy comparison across nutrients.^26^ However, research has found that consumers have trouble understanding and using percentages on food labels.^47, 48, 49, 50^ One solution proposed by the Center for Science in the Public Interest is the inclusion of high/med/low text next to nutrient amounts to aid understanding of the percentage of daily values.^55^ The inclusion of such text has been found to improve FOP label understanding, especially among groups of lower socioeconomic status and education levels.^49^ In FDA online educational materials (see Figure 1), consumers are informed that <5% of a nutrient is ‘low' and >20% of a nutrient is ‘high.' These criteria could also be used as the basis for text indicators and/or text could replace percentages entirely. FDA online materials also use different colors and text to educate consumers about the nutrients that should be limited (for example, total fat, cholesterol, sodium) and those consumers must ‘get enough of' (for example, dietary fiber, vitamins).^56^ These kind of text labels might further aid NFP comprehension and should be studied. NFP clarity might also be improved by sacrificing technical accuracy to communicate more effectively with the consumer. For example, ‘dietary fiber' could be listed as ‘fiber'^55^ and ‘sodium' as ‘salt.' Overall, more research is needed to identify strategies to communicate complicated nutrition information to consumers in meaningful ways, rather than relying exclusively on numeric data (for example, kcal, grams, milligrams, percentages). This is especially important given that those with low literacy and/or numeracy skills have particular difficulty comprehending the NFP.

## FOP nutrition labels

### Improving FOP labeling systems

**Figure 2:**
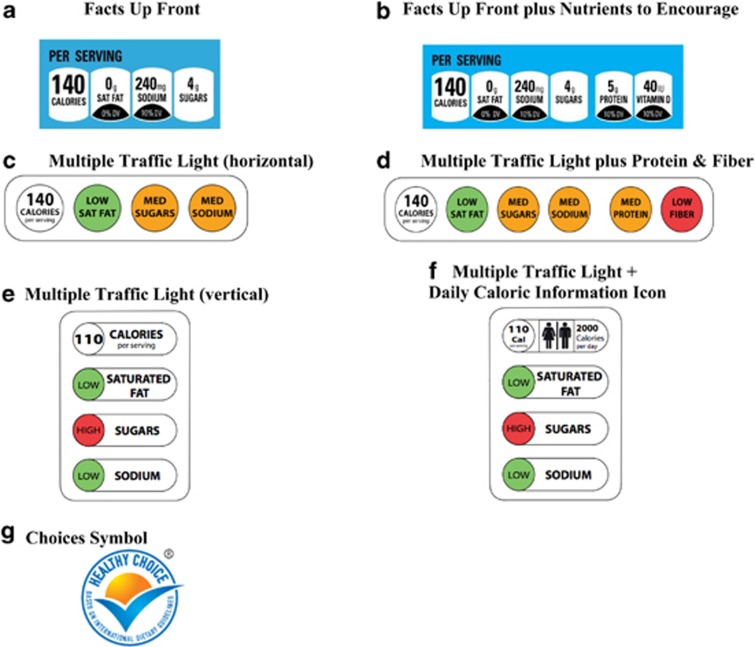
Different FOP nutrition labels. (**a**) Facts Up Front. (**b**) Facts Up Front plus Nutrients to Encourage. Republished here with permission from the Grocery Manufacturers Association. http://www.gmaonline.org/. Available from: http://www.fmi.org/industry-topics/health-wellness/facts-up-front. Accessed June 2013. (**c**) Multiple Traffic Light (horizontal). (**d**) Multiple Traffic Light plus Protein and Fiber. (**f**) Multiple Traffic Light+Daily Caloric Information icon. (**e**) Multiple Traffic Light (vertical). Republished here with permission from the British Heart Foundation. http://www.bhf.org.uk/. Available from: http://www.cdc.gov/pcd/issues/2012/12_0015.htm. Accessed June 2013. (**g**) Choices symbol. Republished here with permission from the Choices Programme. http://www.choicesprogramme.org/. Available from: http://www.cdc.gov/pcd/issues/2012/12_0015.htm. Accessed June 2013.

FOP nutrition labels that display key information in an easy-to-understand format have been proposed as one solution to address the limitations of the NFP and its difficulty capturing consumer attention. Countries worldwide have implemented or are considering implementing different FOP labeling systems (see Figure 2 for sample FOP labels). The Netherlands has adopted the Choices logo, which is a single summary checkmark symbol that appears on products meeting certain standards for low levels of sodium, added sugar, saturated fat, *trans* fat and caloric content.^57^ Fiber and portion size are also considered when appropriate for the group of products. In the United Kingdom, a Multiple Traffic Light labeling system that uses red, yellow and green symbols to alert consumers to low/med/high levels of saturated, fat, sodium and sugar per serving appears on some food products.^58^

The advantage of a Traffic Light labeling system is that it moves beyond traditional information-based approaches by interpreting complicated numeric information for the consumer and harnesses the power of automatic associations between red and ‘stop' and green and ‘go.'^59^ Australia has also recently announced the adoption of a Health Star Rating system, where healthier foods receive more stars, which the food industry has 2 years to voluntarily adopt.^60^ In contrast, FOP labels on products in the United States are not mandated or standardized. This has led to a confusing array of FOP labels developed by different entities.^61^ Several years ago, the FDA announced an initiative to address the lack of a uniform FOP labeling system. As part of these efforts, the Institute of Medicine prepared two reports on the topic that recommended an interpretive, graded symbol that awards food and beverages 0–3 points based on levels of saturated and *trans* fat, sodium and added sugars.^22,23^ It was also recommended that kcals be listed in household measure serving sizes. A review of the extant research literature suggested that FOP labels hold promise as a way to improve consumer understanding of nutrition information and encourage healthier food purchases.^62^

The most recent voluntary industry attempt at a uniform FOP labeling system in the United States has been the Facts Up Front label introduced by the Grocery Manufacturers Association and the Food Marketing Institute. This label displays nutrition information per serving for kcals, saturated fat, sodium and sugars. Manufacturers who voluntarily adopt this scheme can also choose to highlight two ‘nutrients to encourage', such as fiber, potassium or vitamin A.^63^ From a health communications perspective, the design of the Facts Up Front label raises some concerns. The symbol contains a lot of confusing numeric information, including grams and milligrams and percentage of daily values. In addition, it is small and monochrome and does not include any interpretive text.

One Internet-based study examined consumer understanding of different versions of the Facts Up Front symbol relative to versions of the UK's Traffic Light label.^64^ Seven hundred and three adult participants were randomized to either a no label control group or one of the four FOP labels. Two versions of the Traffic Light label were tested. Both included kcal per serving and text (high/med/low) indicating amounts of saturated fat, sodium and sugars per serving, but one version also had information about protein and fiber. Two versions of the Facts Up Front label were tested as well, one of which displayed information about nutrients to encourage (for example, vitamins, protein, fiber). Participants briefly viewed a public service announcement about each labeling system and then completed a quiz asking them to identify which of two products was higher or lower in different nutrient amounts. The Traffic Light and Facts Up Front labels that included nutrients to encourage performed the best on the nutrient comparison quiz. However, when asked to evaluate the nutrient profile of individual products, those viewing Traffic Light labels far outperformed the other label groups, while those who viewed Facts Up Front labels were more likely to underestimate the amounts of saturated fat and sugar. Another similar Internet-based study found that a Traffic Light label that was augmented by an icon of male/female figures and the text ‘2000 calories per day' further improved consumer understanding of nutrition information relative to a Traffic Light label without the 2000 calorie text.^65^ Such icons might be useful, because they provide information that puts calories per serving in context. Another possibility is that the inclusion of a graphic with male/female figures did a better job capturing consumer attention.

The beverage industry has also launched their Clear on Calories initiative, which displays FOP labels with kcals per container.^66^ However, total kcals per container is only displayed on bottles that are ⩽20 oz.; those >20 oz. display kcals per serving and differ depending on whether the drink is a juice (calories are listed per 8 oz. serving) or other beverage (calories are listed per 12 oz. serving).

One study by Vanderlee *et al.*^67^ randomized 687 Canadian consumers to a Coca-Cola bottle that displayed either an FOP label or a nutrition facts table with kcals per serving or kcals per container. Across study groups, 54.2% of participants correctly identified kcals in the entire container, while 35.8% underestimated them. People who saw kcals per container labels versus per serving were significantly more likely to correctly estimate the kcals per container. One limitation was that the sample population was well-educated, limiting the ability to generalize the study findings. More research is needed to understand the influence of the Clear on Calories labels and whether consumer knowledge as well as behavior is influenced when kcals are presented for the entire bottle versus per serving, even when the bottle is >20 oz.

### Serving size information on FOP labels

FOP labels might also represent an opportunity to educate consumers about appropriate serving sizes, but many FOP labeling systems do not present serving size information. Few studies have been conducted to examine how serving size information on an FOP label might influence consumer perceptions and behavior. In one lab-based study, participants were invited to try a cereal for breakfast.^40^ Two hundred and sixteen participants were randomized to one of the three FOP labels based on the Smart Choices FOP labeling system briefly introduced on some food products in the United States in 2009. The rectangular symbol included the words ‘Smart Choices' along with a check mark and information about kcals per serving and servings per package. The three FOP label conditions were: (1) no label control group; (2) the Smart Choices label with the text: 120 calories per serving, 11 servings per package; or (3) the Smart Choices label with the text: 120 calories per ¾ cup serving and 11 servings per package. Participants answered focus group questions about their perceptions of the cereal and poured and ate it for breakfast.

There were no differences in the amount of cereal and milk poured and consumed during the breakfast meal. However, across conditions, participants were pouring almost twice the recommended serving on average. The label groups also did not differ in perceptions of cereal taste, healthfulness or likelihood to purchase the cereal. Those who viewed the FOP labels with calorie information were better able to more accurately estimate the kcals per serving than control participants. Although the label had little impact on behavior, improving people's ability to estimate calories has value given research demonstrating people's difficulty estimating the caloric content of foods consumed outside the home.^68,69^ It is possible that the label in this study might have had a limited effect, because the serving size amounts were perceived as unrealistic and not representing what people actually consume. Another possibility is that presenting serving size information in cups or similar measurement units might still be difficult for people to visualize, especially for individuals who cook infrequently. Only one, relatively unhealthy cereal, was tested in this study, and the sample was composed largely of individuals of high socioeconomic status, limiting the generalizability of the findings.

These results suggest that labels with serving size information might not influence food consumption and the inclusion of serving size information might make the label overly complicated. Therefore, future research should examine how serving size information on FOP labels might influence consumer understanding, perceptions and behaviors. Another challenge is to come up with meaningful serving size units that can be easily conveyed on food packaging. Some professional weight loss treatments educate people about serving and portion sizes using familiar everyday objects (for example, a deck of cards represents a 3 oz. serving of meat, a large handful is a cup of dry cereal),^70^ but little research has examined these kinds of strategies on food packaging.

Other possibilities to help people consume smaller portions are to use salient cues that interrupt mindless overeating. For example, Geier *et al.*^71^ found that people ate the least number of potato chips from a can when a red chip appeared every 7 chips compared with a red chip appearing every 14 chips or cans with no red chips at all. Food companies could experiment with package design that has clear indicators of pre-portioned servings. Other ways to help consumers serve appropriate portion sizes might be to have markers on the outside of food packaging that denote serving size amounts (that is, a 20-oz. bottle of soda could have rings around the outside indicating the points at which one has consumed one and then two servings). More experiments on these kinds of portion size indicators would be valuable.

Lots of FOP labels currently exist and should be compared against one another in both lab and field trials. Sales data from supermarkets that have implemented shelf-tag labeling systems as well as data from cafeterias willing to introduce labeling schemes would be especially valuable in determining optimum labeling formats. An additional area for future research is examining how FOP labels/graphics might be designed to influence children's food choices or interactions with parents when shopping. Although most nutrition labels are designed for adults, much of the food marketing with which they compete is child-targeted. Finally, when considering the optimum design for an FOP label, it is important to think about label elements that might promote the greatest industry reformulation of products. Data on the Choices logo in the Netherlands suggests that the introduction of the symbol encouraged reformulation of food products and the introduction of healthier foods and beverages.^72^ Single-summary logos or interpretive symbols, such as Traffic Lights or health stars, would likely promote greater reformulation than labels like Facts Up Front that lack a clear evaluative component to help consumers interpret the numeric information.

### Nutrition labeling of restaurant meals

#### Menu labeling

The most significant step in nutrition labeling of restaurant foods has been the pending nation-wide introduction of menu labeling, which is part of the Patient Protection and Affordable Care Act.^24^ Menu labeling requires chain restaurants to post calorie information for entire food items at the point-of-purchase. Research on the influence of menu labeling on consumer purchases is mixed, with some studies showing no effect of menu labeling^73, 74, 75^ and others finding that labeling encouraged reductions in kcals purchased and/or consumed.^76, 77, 78, 79, 80, 81, 82^ Menu labeling is a major step forward to educate the public about kcals in restaurant food, which people have great difficulty estimating.^68,69^ However, in its current form it relies on presenting numeric information to inform and/or influence food choices. Given the mixed findings on menu labeling, newer research is examining ways to maximize its effectiveness. One randomized, controlled lab-based study found that adults viewing calorie labels on menus during a dinner meal ordered and ate fewer kcals at the meal.^78^ However, the inclusion of a label on the menu that placed calorie information in context by indicating that the recommended daily caloric intake for adults is 2000 kcal prevented participants from eating more after a dinner meal. Menus with calorie labels, but no contextual label, did not have this effect. This highlights the importance of anchoring caloric information and, in general, making numeric nutrition information more meaningful by putting it in contexts consumers can more easily understand.

The future of nutrition labeling research should be focused on developing and testing numeric and non-numeric ways to more effectively convey nutrition information. One example is a study conducted in a hospital cafeteria, which found that a Traffic Light labeling system promoted purchases of green, healthier items and decreased purchases of red, less healthy items.^83^ Those with lower education levels also benefited most from the Traffic Light labeling system.^84^ The impact of restaurant calorie labels might also be improved by overlaying Traffic Lights to denote lower calorie items or smaller portions and/or by rank-ordering the calories from low-to-high to facilitate information processing.^85^ In addition, Bleich *et al.*^86^ found that presenting calorie information for a sugary drink as an exercise equivalent (50 min to burn a 250-kcal beverage) significantly reduced purchases of sugar-sweetened beverages among adolescents. The increased use of digital menu boards at fast-food chain restaurants would allow for easier implementation and experimentation with different nutrition label formats.

Although most studies on menu labeling have not examined specific influences on portion size, Vermeer *et al.*^87^ assessed the impact of portion size and Guideline Daily Amount (GDA) on Dutch consumers' (*n*=89) portion size choice and intake of soft drinks while at the movies. They conducted the study on 2 days (one control and one experimental). For the experimental condition (*n*=48), consumers could select between five different portion sizes (200, 250, 400, 500, 750 ml cups that ranged from 0.8 to 3 servings) of a soft drink. The soft drinks were accompanied with portion size information and caloric GDA labels that use percentages to put the calories in the context of the overall daily diet. In the control condition (*n*=41), consumers had the same choice of portion sizes but only got ml information. In all, 37.5% of the consumers chose the 250- or 200-ml cups, but labeling did not impact portion size decisions or the amount of liquid consumed. However, the study was limited by the offering of free beverages and a small sample with a limited number of regular soft drink consumers. Nonetheless, the results suggest that offering smaller portions is more effective than trying to use the percentage GDA labeling to reduce portion size choices.

#### Other restaurant labeling strategies

There are also other labeling strategies that could be leveraged to influence decisions about portion size. Ayindoglu and Krishna^88^ conducted a series of five experimental studies to evaluate the impact of qualitative size labels (small, medium, large) on size estimation and consumption of food. Across the five studies, between 58 and 82 university students were recruited and presented with different servings of snacks (pretzels, nuts, sandwiches, cookies) that were accompanied by various size labels. When a larger food item was labeled ‘down' toward a smaller size, consumers perceived the food amount to be less. The perceived amount consumed from a package labeled ‘small' was also lower than the amount actually eaten; these effects were more marked when people were under competing cognitive demands. In addition, participants who were given a snack labeled ‘medium size' ate more than those given the same snack labeled ‘large size.' Additionally, provision of information on serving size did not lessen the effect of size labels. That is, large sizes were perceived as small if they were labeled small, even in the presence of serving size information in grams. However, consumers concerned about their health were less likely to rely on size labels.

This study revealed that consumers will continue to eat large amounts of food when a label is switched from ‘large' to ‘small,' but they will feel that they have not eaten too much, a phenomenon the authors call ‘guiltless gluttony.'^88^ A study conducted by Just *et al.*^89^ found similar results. They used prepared foods (spaghetti and salad) and found that consumers wasted more food when a large portion was called a ‘double-size' than when the large portion was called ‘regular'. Similarly, individuals left more salad on their plate when it was labeled ‘regular' versus ‘half-size.' These results suggest that there might be promising opportunities for restaurants to experiment with differentially labeling healthy foods with smaller size labels to promote increased consumption, while serving smaller portions of less healthy foods but labeling them as ‘large.' Such labels might also be leveraged for packaged foods typically consumed in one sitting, rather than presenting numeric serving size information.

### Summary of recommendations to improve nutrition labels

Updating the NFP, developing uniform FOP labeling symbols and providing consumers with nutrition information on restaurant menus offer important opportunities to educate people about the nutritional content of their food and motivate consumers to make healthier choices. Although government agencies have worked to design easy-to-understand nutrition labeling systems, there is always room for improvement based on scientific advances. Requiring the NFP on packaged foods in the United States was a major step forward in informing consumers and making people aware of the importance of nutrition. However, much of the nutrition information presented to the public has taken the form of numeric data, some of which requires mathematical manipulation to use effectively.

Future research on nutrition labeling should focus on designing better numeric and non-numeric strategies to convey nutrition information to the public through the NFP, FOP labels and menu labeling. These efforts should focus on ways to improve food labels' ability to capture consumer attention, reduce the complexity of labels and identify ways to convey nutrition information in meaningful units. Current efforts to update the NFP should specifically focus on addressing confusion around serving size. Research suggests that consumers would benefit from the NFP and FOP labels displaying nutrition information for an entire container for those foods and beverages typically consumed in one sitting. Efforts should also be made to standardize serving sizes for these items. In addition, data are needed to determine whether serving sizes should continue to be based on the original RACC or should be updated to match current consumption norms. Additional ways to improve and/or supplement labeling should be tested further, including designing food packaging with salient cues that alert consumers to serving size amounts, adding text to food labels to improve understanding of numeric data and examining non-numeric strategies to convey nutrition information on packaged and restaurant foods.

Accurate and easy-to-understand nutrition labeling is a worthy public health goal that should be considered an important strategy among many to address obesity and poor diet. At a minimum, labeling provides consumers with information they are entitled to, and as labeling interventions are being pursued, they should be implemented in the most useful and cost-effective manner. Even if food labeling results in only small changes in caloric or other nutrient intake, this can lead to meaningful change on a population level.^90^ Modeling studies also suggest that nutrition labeling strategies, such as FOP labels on packaged foods, are more cost-effective than other interventions and treatments for obesity.^86^ Finally, well-designed labels have the potential to ‘nudge'^91^ consumers by altering the context in which people make decisions about food choices and consumption without limiting those choices or altering economic incentives. However, labels also have the potential to ‘nudge' the food industry to reformulate foods and offer healthier alternatives, which might be the most powerful impact of labeling interventions.
